# Longitudinal change in arterial stiffness after delivery in women with preeclampsia and normotension: a prospective cohort study

**DOI:** 10.1186/s12884-020-03374-0

**Published:** 2020-11-11

**Authors:** Sehun KIM, Hyun Ja LIM, Jeung-Ran KIM, Kyung Joon OH, Joon-Seok HONG, Jung-Won SUH

**Affiliations:** 1 Department of Internal Medicine, Seongnam Citizens Hospital, Seongnam, Republic of Korea; 2 Department of Community Health & Epidemiology, College of Medicine, University of Saskatchewan, Saskatoon, Canada; 3 Department of Internal Medicine, Cardiovascular Center, Seoul National University Bundang Hospital, Seoul National University College of Medicine, 82 Gumi-ro, 173 Beon-gil, Bundang-gu, Seongnam, 13620 Republic of Korea; 4 Department of Obstetrics and Gynecology, Seoul National University Bundang Hospital, Seoul National University College of Medicine, 82 Gumi-ro, 173 Beon-gil, Bundang-gu, Seongnam, 13620 Republic of Korea

**Keywords:** Blood pressure, Cardiovascular diseases, Cardio-ankle vascular index, Longitudinal change, Preeclampsia, Pregnancy, Vascular stiffness

## Abstract

**Background:**

Preeclampsia is associated with increased arterial stiffness during pregnancy. However, data on the longitudinal change in arterial stiffness after delivery in women with preeclampsia are lacking. In this pilot study, we aimed to examine the longitudinal change in arterial stiffness using the cardio-ankle vascular index after delivery in women with preeclamptic and normotensive pregnancies.

**Methods:**

We enrolled pregnant women with preeclampsia (*n* = 37) and normotension (*n* = 36) who gave birth at Seoul National University Bundang Hospital between March 2013 and May 2016, and followed-up at day 1, 6 months, and 12 months after delivery. The longitudinal change in the cardio-ankle vascular index and other variables (blood pressure, lipid profiles, serum creatinine, and liver enzymes) were compared between the two groups using the mixed-effects model, and interactions among the main predictors were examined.

**Results:**

The longitudinal change in the cardio-ankle vascular index did not significantly differ between the two groups (β = 0.11, 95% CI: − 0.31–0.54, *p* = 0.60). Predictors of the longitudinal change in the cardio-ankle vascular index included age, time since delivery, body mass index, and diabetes mellitus. Women with preeclampsia showed significantly elevated blood pressure, lipid profiles, serum creatinine, and liver enzymes compared to women with normotension over the course of 1 year of follow-up.

**Conclusions:**

Preeclampsia is associated with unfavorable blood pressure and metabolic indices after delivery. However, we found no difference in the longitudinal change in arterial stiffness between women with preeclampsia and normotension over the course of 1 year after delivery.

**Trial registration:**

Retrospectively registered at ClinicalTrials.gov on October 29, 2019 (NCT04142268).

## Background

Hypertensive pregnancy disorders, such as preeclampsia or eclampsia, are one of the leading causes of maternal mortality during pregnancy and the puerperium, affecting 2–8% of gestations [1–3]. These disorders are associated with vascular endothelium dysfunction, insulin resistance, hyperlipidemia, hypercoagulability, and inflammation [4–6]. Thus, hypertensive pregnancy disorders share many etiologies with cardiovascular disease. There is accumulating evidence that women with a history of hypertensive pregnancy disorders have increased risks of cardiovascular disease [7, 8].

Arterial stiffening develops from a complex interaction between stable and dynamic changes in the structural and cellular elements of the vessel wall [9]. Furthermore, arterial stiffening is a marker for increased cardiovascular risks such as myocardial infarction, heart failure, and total mortality [10]. Previous studies suggest that women with preeclampsia have increased arterial stiffness during pregnancy compared to pregnant women with normotension [11, 12]. However, there are few reports on the longitudinal change in arterial stiffness after delivery in women with preeclampsia.

In the present pilot study, we aimed to longitudinally follow arterial stiffness, as assessed by the cardio-ankle vascular index (CAVI), blood pressure (BP) and other metabolic indices, for 1 year after delivery. Furthermore, we investigated the predictive markers of increased postpartum arterial stiffness.

## Materials and methods

### Study participants

This prospective cohort study included 37 women with preeclampsia and 36 women with normotension who gave birth at Seoul National University Bundang Hospital (SNUBH) between March 2013 and May 2016. SNUBH is a teaching and tertiary referral hospital that provides care for high-risk deliveries. Women ages 18 to 45 were eligible for the study. We excluded women with a pregnancy that ended in stillbirth, and those who were hemodynamically compromised or had peripartum bleeding complications.

The diagnosis of preeclampsia was made based on the criteria of the International Society for the Study of Hypertension in Pregnancy. Under this classification, preeclampsia is defined as diastolic BP of at least 110 mmHg on one occasion or diastolic BP of at least 90 mmHg on two consecutive occasions more than 4 h apart, in combination with proteinuria (≥300 mg total protein in a 24-h urine collection or, if this is not available, ≥2+ proteinuria by dipstick analysis on two consecutive occasions at least 4 h apart) that develops after 20 weeks of gestation in women who were previously normotensive [13].

Study participants were treated at the physician’s discretion according to current recommendations and guidelines for the peripartum and postpartum periods.

### Study protocol

The CAVI is calculated based on the stiffness parameter, and is theoretically independent of changes in BP. Because of this distinct advantage, the CAVI has been applied clinically to assess arterial stiffness in patients with known cardiovascular diseases (atherosclerosis, coronary heart disease, and stroke), as well as in those at risk for cardiovascular diseases (hypertension, diabetes, obesity, and advanced age) [14–16].

The CAVI was measured on day 1, 6 months, and 12 months after delivery in both study groups.

The body mass index (BMI), BP, lipid profiles, serum creatinine, aspartate transaminase (AST), and alanine transaminase (ALT) levels were assessed simultaneously with the CAVI.

### Laboratory measurements

Blood samples were collected from the antecubital vein after 8–10 h of fasting. Laboratory performance was monitored regularly by a quality control program. Hemoglobin was measured using the XE-2100 D (Sysmex Inc., Kobe, Japan). Serum creatinine, ALT, AST, total cholesterol, high-density lipoprotein (HDL) cholesterol, low-density lipoprotein (LDL) cholesterol, and triglyceride (TG) levels were measured using the Beckman Coulter AU 5800 analyzer (Beckman Coulter Inc., Brea, CA, USA).

### Measurement of BP and the CAVI

After the study participant had rested in a seated position for at least 5 min, BP was measured three times on the right arm, using an appropriately sized arm cuff and validated upper arm BP monitor (HEM-7200; Omron healthcare Co., Kyoto, Japan) [17]. The final B*P* value was obtained by averaging the second and third measurements. Height and body weight were measured during each visit using standardized equipment (G-310c, G-Tech Co., Uijeongbu, South Korea).

The CAVI was measured in the supine position using a Vasera VS-1000 vascular screening system (Fukuda Denchi, Tokyo, Japan). The principles of the CAVI have been described in previous reports [16, 18]. The automatically obtained data from the right and left CAVIs were analyzed using VSS-10 software. The average values of the right and left CAVIs were used for analysis.

### Statistical analysis

Data distribution was examined and checked for potential outliers. Descriptive statistics were used to summarize the baseline demographic and clinical characteristics of the study participants. Group differences were evaluated using a t-test or the Wilcoxon test for continuous variables, and the Chi-square test or Fisher’s exact test for categorical variables.

The relationship between the presence of preeclampsia and longitudinal changes in the CAVI, with and without control for other potential covariates, was examined using a mixed-effects model with compound symmetry covariance structure to adjust for repeated measurements within participants. The mixed-effects model accounted for all available data points, therefore, respondents with incomplete datasets were not excluded from analysis (under the assumption that missing data occurred at random). Interactions among the main predictors in the final model were examined. For the mixed-effects model, R^2^ as the proportion of explained variance can be categorized into two types: marginal R^2^ (R^2^_(m)_) and conditional R^2^ (R^2^_(C)_). R^2^_(m)_ is concerned with variance explained by fixed effects. R^2^_(C)_ is concerned with variance explained by both fixed effects and random effects. Thus, differences between corresponding R^2^_(m)_ and R^2^_(C)_ values reflect how much variability is in random effects [19, 20]. Group differences were also evaluated across the reporting time points for several other physiological parameters of interest using mixed-effects modeling.

All reported *p*-values are 2-tailed, and α = 0.05 was set as a threshold for statistical significance. All statistical analyses were performed using SAS version 9.4 (SAS Institute, Cary, NC, USA).

## Results

### Study flow

**Fig. 1:**
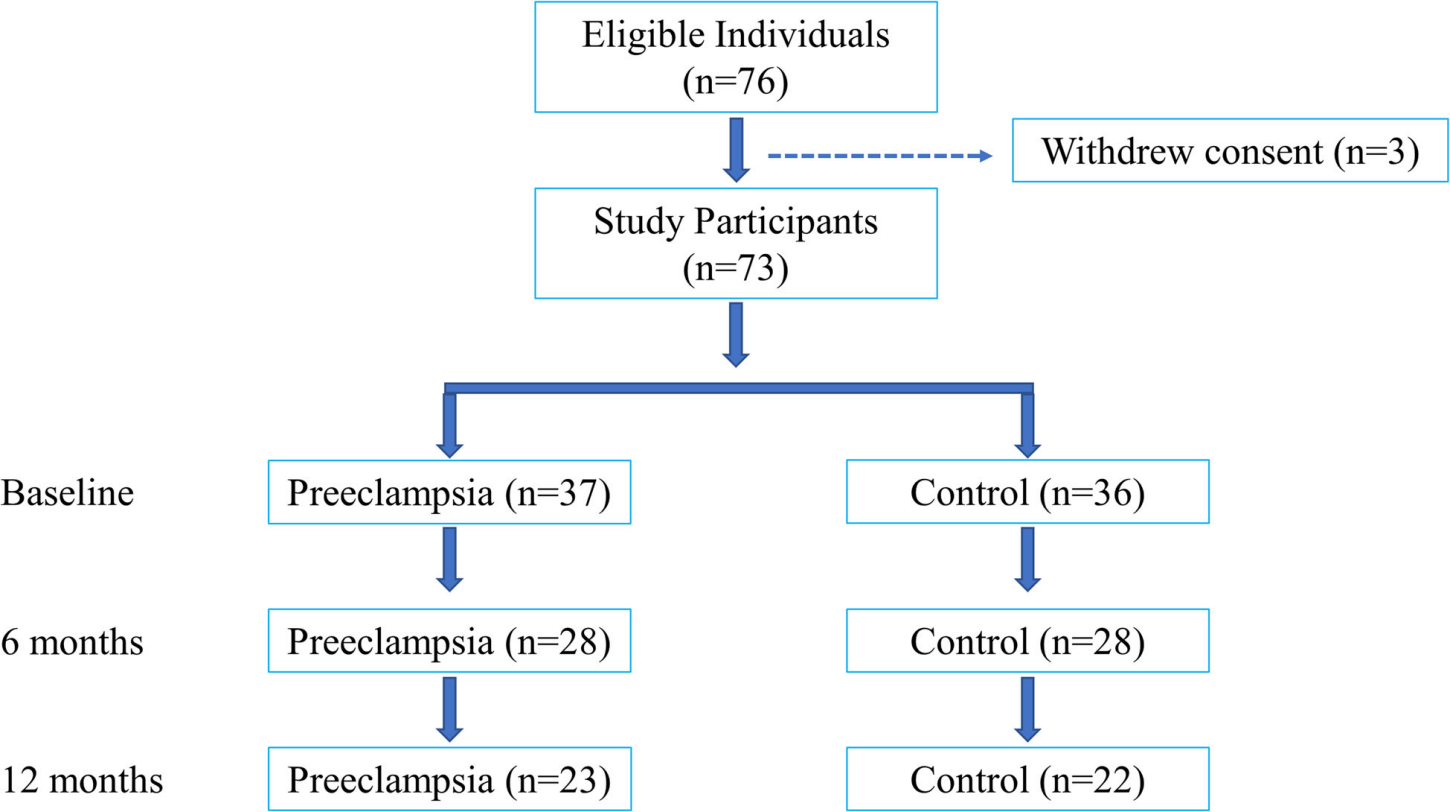
Study Flow Chart

The study flow is depicted in Fig. 1. The overall follow-up rate was 76.7% (*n* = 56) and 61.6% (*n* = 45) at 6 and 12 months, respectively. There was no difference in the follow-up loss rate at 1 year between the two groups (*p* = 0.81). There were no clinical events in either group such as death, myocardial infarction, or hospitalization from heart failure or renal deterioration. Participants who dropped out of the study indicated that they did not have time to participate due to infant care. CAVI measurements were performed in 71 women (97.2%) at baseline, 50 women (68.5%) at 6 months, and 45 women (61.6%) at 12 months.

### Baseline characteristics

The baseline characteristics of the study participants are shown in Table 1. There was no significant difference in the baseline mean CAVI between the control and preeclampsia groups (6.92 ± 1.69 vs 6.53 ± 1.5, *p* = 0.306). However, compared to those in the control group, the women in the preeclampsia group were significantly older (34.8 ± 3.6 years vs 32.9 ± 3.6 years, *p* = 0.029), and had significantly higher systolic BP (142.4 mmHg vs 111.4 mmHg *p* < 0.001) and diastolic BP (83.9 mmHg vs 63.2 mmHg, *p* < 0.001). There were also significant differences between the two groups in the baseline levels of total cholesterol, triglyceride, creatinine, AST, and ALT. Furthermore, 62.2% (23/37) of the women with preeclampsia were treated with antihypertensive drugs. A calcium-channel blocker (*n* = 23, 100%) was the most prescribed antihypertensive agent; however, two women were also prescribed beta-blockers. In patients with preeclampsia, 22 had early-onset preeclampsia (defined as preeclampsia that develops before 34 weeks of gestation), whereas 15 had late-onset preeclampsia.

**Table 1:** Baseline demographic and clinical characteristics

Variable	Preeclampsia (***n*** = 37)	Control (***n*** = 36)	***P*** value^**a**^
Age, years	34.8 ± 3.6	32.9 ± 3.6	0.029
History of preeclampsia, n (%)	2 (5.4)	1 (2.8)	0.999
Onset of preeclampsia, n (%)			
Early onset	22 (59.5)		
Late onset	15 (40.5)		
Peak body weight during pregnancy, kg	70.4 ± 11.8	65.6 ± 7.5	0.042 (0.108^b^)
Primipara	24 (64.9)	24 (66.7)	0.871
Twins, n (%)	4 (10.8)	1 (2.8)	0.358
Gestational week at delivery	33.7 ± 3.0	37.7 ± 2.0	<0.001
Fetal birth weight, kg	1.79 ± 0.58	2.93 ± 0.52	<0.001
Waist-to-hip ratio	0.92 ± 0.05	0.92 ± 0.06	0.874
BMI, m^2^/kg	27.4 ± 5.0	25.0 ± 2.8	0.015 (0.050^b^)
Mean CAVI^c^	6.92 ± 1.69	6.53 ± 1.50	0.306 (0.032^b^)
Hypertension^d^, n (%)	2 (5.4)	0	0.494
Diabetes mellitus^e^, n (%)	4 (10.8)	0	0.115
SBP, mmHg	142.4 ± 12.7	111.4 ± 10.5	< 0.001
DBP, mmHg	83.9 ± 9.3	63.2 ± 7.8	< 0.001
Hemoglobin, mg/dL	12.0 ± 2.0	11.4 ± 1.8	0.164
Total cholesterol, mg/dL	232.6 ± 49.6	208.6 ± 46.3	0.036
HDL cholesterol, mg/dL	59.9 ± 14.6	62.6 ± 13.1	0.419
LDL cholesterol, mg/dL	126.2 ± 33.0	112.2 ± 33.7	0.082 (0.017^b^)
Triglyceride, mg/dL	255.6 ± 92.5	213.6 ± 60.5	0.026 (0.061^b^)
Creatinine, mg/dL	0.693 ± 0.255	0.532 ± 0.092	0.001 (0.007^b^)
AST, IU/L	31.0 ± 17.2	17.9 ± 7.6	< 0.001
ALT, IU/L	27.0 ± 26.9	11.4 ± 6.2	0.002 (<0.001^b^)
Hypertension treatment, n (%)	23 (62.2%)	0%	<0.001
Treatment regimen, n (%)			
Single drug	13 (56.5)		
Two drugs	8 (34.8)		
Three drugs	2 (8.7)		
Type of antihypertensive drug, n (%)			
CCB	23 (100%)		
BB	2 (8.7%)		

Data are presented as numbers and percentages or mean ± SD. ^a^T-test or Chi-square test. ^b^The Mann-Whitney test was used due to a skewed distribution. ^c^The mean CAVI is the average of the right and left CAVIs. ^d^refers to pre-pregnancy hypertension. ^e^Two women had type-1 diabetes mellitus; the remaining women had type-2 diabetes mellitus

*SD* Standard deviation, *BMI* Body mass index, *CAVI* Cardio-ankle vascular index, *SBP* Systolic blood pressure, *DBP* Diastolic blood pressure, *HDL* High density lipoprotein, *LDL* Low density lipoprotein, *AST* Aspartate aminotransferase, *ALT* Alanine aminotransferase, *CCB* Calcium channel blocker, *BB* Beta blocker

### Relationship between preeclampsia and longitudinal change in the CAVI

**Fig. 2:**
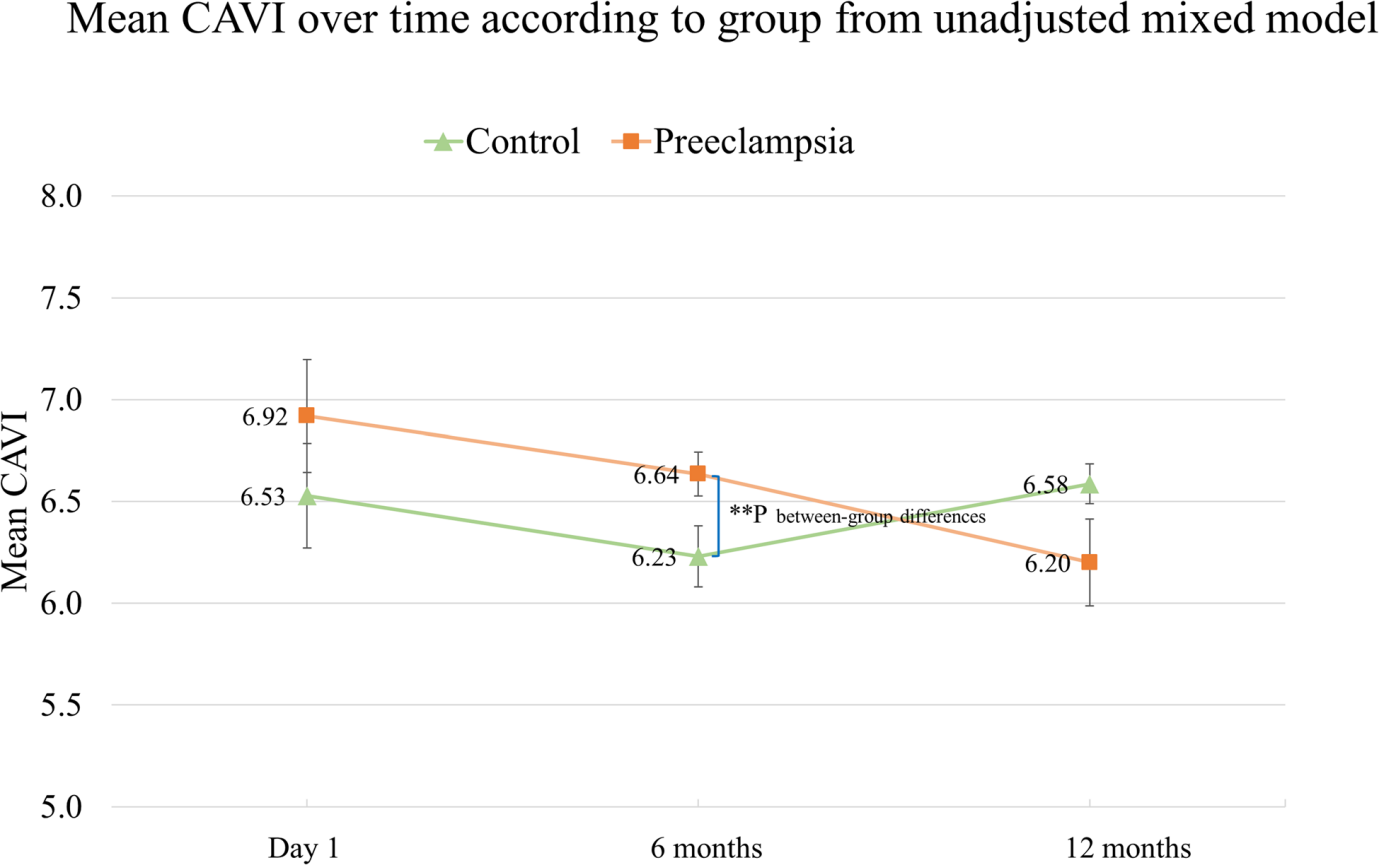
Mean CAVI over time according to group from unadjusted mixed modeling. Data are presented as the mean level ± SE, and significant differences are indicated by double asterisks (** *p* < 0.05). CAVI, cardio-ankle vascular index; SE, standard error

Both between-subject (group) and within-subject (time) differences in the mean CAVI were analyzed. At 6 months, the mean CAVI was higher in the preeclampsia group than in the control group (6.64 vs 6.23, *p* = 0.03). However, at 12 months, the group difference did not remain significant (Table 2, Fig. 2). For the preeclampsia group, the mean CAVI showed a decreasing trend over 1 year; however, the trend was not statistically significant. For both groups, the within-subject comparisons in the mean CAVI between time points did not reveal any significant differences (Table 3, Fig. 2).

**Table 2:** Between-subject (group) differences in the mean CAVI^a^ at each time point

	Time	Control	Preeclampsia	***P***-value
**Mean CAVI**	Day 1	6.53 (6.01, 7.05)	6.92 (6.36, 7.48)	0.31
	6 months	6.23 (5.91,6.55)	6.64 (6.41, 6.86)	0.03
	12 months	6.58 (6.38, 6.79)	6.20 (5.75, 6.65)	0.09

Data are presented as mean and 95% C.I. ^a^The mean CAVI is the average of the right and left CAVIs

*CAVI* Cardio-ankle vascular index, *C. I* Confidence interval

**Table 3:** Within-subject (time) differences in the mean CAVI^a^, with comparisons between pairs of time points

	Group	Day 1	6 months	12 months	***P***-value
**Mean CAVI**	Control	6.52 (6.15, 6.91)	6.23 (5.74, 6.72)	–	0.61
		6.52 (6.15, 6.91)	–	6.59 (6.08, 7.09)	0.98
			6.23 (5.74, 6.72)	6.59 (6.08, 7.09)	0.57
	Preeclampsia	6.92 (6.51, 7.33)	6.64 (6.15, 7.12)	–	0.64
		6.92 (6.51, 7.33)	–	6.20 (5.57, 6.83)	0.14
		–	6.64 (6.15, 7.12)	6.20 (5.57, 6.83)	0.52

Data are presented as mean and 95% C.I. ^a^The mean CAVI is the average of the right and left CAVIs

*CAVI* Cardio-ankle vascular index, *C. I* Confidence interval

### Predictors of the longitudinal change in the CAVI

After adjusting for the time since delivery, age, BMI, and diabetes mellitus, there was no difference in the longitudinal change in the mean CAVI between the two groups (β = 0.11, 95% confidence interval [C.I]: − 0.31 – 0.54, *p* = 0.60). No interaction between group and time was observed in this mixed-effects model (*p* = 0.2488).

The mixed-effects model indicated that the time since delivery (β = − 0.54, 95% C.I: − 0.97 – - 0.11, *p* = 0.015), presence of diabetes mellitus (β = 1.07, 95% C.I: 0.25–1.89, *p* = 0.012), age (β = 0.08, 95% C.I: 0.02 – 0.14, *p* = 0.013), and BMI (β = − 0.07, 95% C.I: − 0.12 – 0.03, *p* = 0.003) were significant predictors of the longitudinal change in the CAVI. No interactions were observed among any of the covariates in the mixed-effects model (Table 4).

**Table 4:** Predictors of the longitudinal change in the mean CAVI^a^ from mixed-effects modeling

	β estimate ± SE	95% C.I	***P***-value
Time since delivery:			
Day 1	–	–	
6 months	− 0.54 ± 0.22	− 0.97, − 0.11	0.015
12 months	− 0.62 ± 0.24	−1.10, − 0.14	0.012
Group:			
Control	–	–	
Preeclampsia	0.11 ± 0.21	− 0.31, 0.54	0.600
BMI	− 0.07 ± 0.02	− 0.12, 0.03	0.003
Age	0.08 ± 0.03	0.02, 0.14	0.013
Diabetes Mellitus:			
No	–	–	
Yes	1.07 ± 0.41	0.25, 1.89	0.012
*R*^*2*^_*(m)*_ *= 0.1528, R*^*2*^_*(C)*_ *= 0.3624*			

^a^The mean CAVI is the average of the right and left CAVIs. Interactions between covariates in the final model were examined and none were found to be statistically significant

*R*^*2*^_*(m)*_ marginal R^2^, *R*^*2*^_*(C)*_ conditional R^2^, *SE* Standard error, *C. I* confidence interval, *BMI* Body mass index

In addition, when R^2^ was considered as the proportion of explained variance, substantial differences between marginal R^2^ and conditional R^2^ in the mixed-effects models were observed. This indicates that a substantial amount of variability was in random effects.

### Relationships between preeclampsia and BP/metabolic indices

Compared to the control group, the preeclampsia group had significantly higher age- and time-adjusted levels of systolic BP (β = 21.6, 95% C.I: 15.9–27.4, *p* < 0.0001), diastolic BP (β = 16.1, 95% C.I: 12.0–20.2, *p* < 0.0001), waist/hip ratio (β = 0.04, 95% C.I: 0.01 – 0.07, *p* = 0.016), total cholesterol (β = 20.5, 95% C.I: 2.08–38.8, *p* = 0.03), triglyceride (β = 31.7, 95% C.I: 0.41–62.9, *p* = 0.047), creatinine (β = 0.115, 95% C.I: 0.032–0.197, *p* = 0.007), AST (β = 4.78, 95% C.I: 1.22–8.35, *p* = 0.009), and ALT (β = 7.23, 95% C.I: 1.53–12.9, *p* = 0.014) during follow-up (Table 5). However, no group differences were observed in the LDL cholesterol, and HDL cholesterol.

**Table 5:** Impact of preeclampsia on longitudinal changes in blood pressure and metabolic indices

Outcome	Covariates	β estimate ± SE	95% C.I	***P***-value
Systolic BP (mmHg)	Time since delivery:			
	Day 1	–	–	
	6 months	−10.6 ± 2.12	−14.8, −6.41	< 0.0001
	12 months	−10.3 ± 2.36	−15.0, −5.60	< 0.0001
	Group:			
	Control	–	–	
	Preeclampsia	21.6 ± 2.90	15.9, 27.4	< 0.0001
	Age	0.46 ± 0.41	− 0.36, 1.28	0.273
	*R*^*2*^_*(m)*_ *= 0.0502, R*^*2*^_*(C)*_ *= 0.4083*			
Diastolic BP (mmHg)	Time since delivery:			
	Day 1	–	–	
	6 months	−2.75 ± 1.46	− 5.66, 0.16	0.064
	12 months	− 0.59 ± 1.63	−3.84, 2.66	0.720
	Group:			
	Control	–	–	
	Preeclampsia	16.1 ± 2.07	12.0, 20.2	< 0.0001
	Age	0.32 ± 0.29	− 0.27, 0.90	0.287
	*R*^*2*^_*(m)*_ *= 0.0343, R*^*2*^_*(C)*_ *= 0.4393*			
Waist/Hip ratio	Time since delivery:			
	Day 1	–	–	
	6 months	− 0.10 ± 0.02	− 0.14, − 0.06	< 0.0001
	12 months	− 0.07 ± 0.02	− 0.11, − 0.04	0.0001
	Group:			
	Control	–	–	
	Preeclampsia	0.04 ± 0.02	0.01, 0.07	0.016
	Age	0.006 ± 0.002	− 0.011, − 0.001	0.012
	*R*^*2*^_*(m)*_ *= 0.0252, R*^*2*^_*(C)*_ *= 0.0498*			
Total cholesterol (mg/dL)	Time since delivery:			
	Day 1	–	–	
	6 months	−36.7 ± 5.69	− 48.1, −25.4	< 0.0001
	12 months	− 45.9 ± 6.36	−58.5, − 33.2	< 0.0001
	Group:			
	Control	–	–	
	Preeclampsia	20.5 ± 9.23	2.08, 38.8	0.030
	Age	− 0.14 ± 1.30	−2.73, 2.44	0.913
	*R*^*2*^_*(m)*_ *= 0.1370, R*^*2*^_*(C)*_ *= 0.5486*			
LDL cholesterol (mg/dL)	Time since delivery:			
	Day 1	–	–	
	6 months	−14.3 ± 4.59	−23.4, −5.14	0.003
	12 months	−20.7 ± 5.15	−31.0, − 10.5	0.0001
	Group:			
	Control	–	–	
	Preeclampsia	51.5 ± 30.8	− 9.82, 112.9	0.099
	Age	− 4.50 ± 4.31	−13.1, 4.09	0.30
	*R*^*2*^_*(m)*_ *= 0.4839, R*^*2*^_*(C)*_ *= 0.9700*			
TG (mg/dL)	Time since delivery:			
	Day 1	–	–	
	6 months	− 119.5 ± 12.5	− 144.3, − 94.7	< 0.0001
	12 months	−127.9 ± 13.8	− 155.5, − 100.3	< 0.0001
	Group:			
	Control	–	–	
	Preeclampsia	31.7 ± 15.7	0.41, 62.9	0.047
	Age	− 0.41 ± 2.28	− 4.96, 4.13	0.857
	*R*^*2*^_*(m)*_ *= 0.0288, R*^*2*^_*(C)*_ *= 0.3366*			
HDL cholesterol (mg/dL)	Time since delivery:			
	Day 1	–	–	
	6 months	− 5.15 ± 1.78	− 8.68, −1.61	0.005
	12 months	− 5.17 ± 1.98	− 9.12, − 1.23	0.011
	Group:			
	Control	–	–	
	Preeclampsia	−1.80 ± 2.73	−7.25, 3.64	0.511
	Age	− 0.23 ± 0.39	−1.01, 0.55	0.558
	*R*^*2*^_*(m)*_ *= 0.0878, R*^*2*^_*(C)*_ *= 0.5112*			
Creatinine (mg/dL)	Time since delivery:			
	Day 1	–	–	
	6 months	− 0.002 ± 0.022	− 0.046, 0.041	0.913
	12 months	0.025 ± 0.024	− 0.024, 0.073	0.310
	Group:			
	Control	–	–	
	reeclampsia	0.115 ± 0.041	0.032, 0.197	0.007
	Age	− 0.01 ± 0.006	− 0.021, 0.002	0.089
	*R*^*2*^_*(m)*_ *= 0.0702, R*^*2*^_*(C)*_ *= 0.6368*			
AST (IU/L)	Time since delivery:			
	Day 1	–	–	
	6 months	− 6.07 ± 2.11	− 10.3, −1.88	0.005
	12 months	− 6.49 ± 2.31	− 11.1, − 1.89	0.006
	Group:			
	Control	–	–	
	Preeclampsia	4.78 ± 1.78	1.22, 8.35	0.009
	Age	− 0.15 ± 0.27	− 0.69, 0.39	0.576
	*R*^*2*^_*(m)*_ *= Not Available* ^a^*, R*^*2*^_*(C)*_ *= Not Available*^a^			
ALT (IU/L)	Time since delivery:			
	Day 1	–	–	
	6 months	− 3.00 ± 2.97	− 9.91, 2.90	0.315
	12 months	− 6.55 ± 3.27	−13.1, − 0.045	0.049
	Group:			
	Control	–	–	
	Preeclampsia	7.23 ± 2.86	1.53, 12.9	0.014
	Age	0.016 ± 0.43	− 0.83, 0.86	0.97
	*R*^*2*^_*(m)*_ *= 0.0087, R*^*2*^_*(C)*_ *= 0.0652*			

^a^There is a case where a variance component is zero or very near zeros in mixed models, and thus *R*^*2*^ cannot be calculated

*R*^*2*^_*(m)*_ marginal R^2^, *R*^*2*^_*(C)*_ conditional R^2^, *SE* Standard error, *C. I* Confidence interval, *BP* Blood pressure, *LDL* Low-density lipoprotein, *TG* Triglyceride, *HDL* High-density lipoprotein, *AST* Aspartate transaminase, *ALT* Alanine aminotransferase

## Discussion

### Principal findings

The difference between the groups at 6 months, i.e. the higher mean CAVI in the preeclampsia group, appeared to be related to the older age of the women and the diabetic status, as the group difference was resolved with adjustment. However, by 1 year post delivery, there was no statistically significant difference in the CAVI between the groups. Both groups showed a statistically significant decline in the CAVI by 6 months after delivery and no subsequent meaningful change. There was no statistical evidence that this improvement was dependent on other factors.

Thus, although women with preeclamptic pregnancies persistently showed worse cardiovascular risk profile trajectories after delivery than women with normotensive pregnancies, the arterial stiffness trajectory over the course of 1 year after delivery did not differ between women with preeclampsia and those with normotension.

### Results

Systemic arterial stiffness undergoes major changes during pregnancy. Previous longitudinal studies of women with normotensive pregnancies have demonstrated that the pulse wave velocity (PWV), an index of arterial stiffness, decreases during the second trimester, increases during the third trimester and delivery, and decreases during the first month postpartum [21]. Another study longitudinally followed the changes in PWV throughout pregnancy and 1 month after delivery in women with normal pregnancies, and showed a similar pattern of change in the PWV [22]. On the other hand, other studies revealed that in patients with pregnancy-induced hypertension, the PWV did not decrease between the first and second trimesters, and markedly increased after delivery [21, 23, 24]. The key finding that is consistent in the present and previous studies is an increased postpartum arterial stiffness parameter from 7 weeks up to 2–3 years postpartum [25]. The present study showed an elevated CAVI up to 6 months postpartum. The timing and persistence of endothelial dysfunction after delivery in women with a history of preeclampsia has not been fully elucidated.

To our knowledge, this study is the first longitudinal report of arterial stiffness using the CAVI, and the results suggest that there are measurable changes in the maternal vasculature during pregnancy.

Preeclampsia is associated with greater and more prolonged postpartum increases in arterial stiffness [22]. However, few studies have compared the postpartum longitudinal change in arterial stiffness between women with preeclamptic and normotensive pregnancies. Most studies utilized a cross-sectional design and estimated arterial stiffness by pulse wave analysis (PWA) [11, 26]. BP is one of the most important contributing factors to PWA [27], and unfavorable arterial stiffness indices in women with a history of preeclampsia may be attributable to a higher incidence of hypertension. Thus, in the present study, we examined the longitudinal change in arterial stiffness for 1 year after delivery using the CAVI, which is less affected by BP than the PWA. We observed a significant group difference in the mean CAVI at 6 months after delivery; however, at 12 months, the group difference was no longer significant. Furthermore, the overall difference between the groups in the longitudinal change in the mean CAVI over the course of 1 year after delivery failed to reach statistical significance. This result suggests that postpartum changes in arterial stiffness in women with preeclampsia are reversible, as is the case for normotensive pregnancies.

### Clinical implications

In the present study, we confirmed that preeclamptic pregnancy is associated with higher postpartum trajectories of age- and time-adjusted BP and metabolic indices. These results are consistent with previous reports from Western countries [28, 29]. These factors may contribute to the development of long-term cardiovascular disease. Thus, the present results suggest that an unfavorable cardiovascular risk profile may contribute to the development of future cardiovascular disease to a greater extent than endothelial damage caused by preeclampsia persisting beyond the postpartum recovery period.

Diabetes mellitus was independently associated with an increased CAVI in the mixed-effect model. Gestational diabetes is associated with cardiovascular disease later in life, and the present results suggest that increased arterial stiffness may play a role. Women with gestational diabetes should be closely followed, even if they do not have a history of preeclampsia.

### Research implications

The study results suggest that meticulous follow-up and strict control of cardiovascular disease risk factors in women with a history of preeclampsia are needed. However, to date, there is limited research evaluating the efficacy of different interventional approaches for addressing postpartum cardiovascular health in women with a history of preeclampsia.

### Strengths and limitations

To the best of our knowledge, we have performed the longest follow-up study of postpartum arterial stiffness. In addition, this is the first study that compared the longitudinal change in the CAVI between postpartum women with preeclamptic and normotensive pregnancies.

The present study has several limitations. First, the sample size was relatively small; consequently, we could not confirm the effect of antihypertensive medication use on the longitudinal change in the CAVI. However, to our knowledge, no other study has been published on the longitudinal CAVI change after delivery in women with preeclampsia. Our study was an exploratory pilot study using a convenient sample from the target population. Second, only 76.7 and 61.6% of women completed the study assessments at 6 and 12 months, respectively. Participants who dropped-out were too busy with childcare to participate in the study. In South Korea, the burden of infant care is usually concentrated on mothers, and support from the welfare system is inadequate [30]. Thus, the assumption that data was missing at random is reasonable, and the use of mixed-effects modeling under this assumption was appropriate. Third, the heterogeneity of the preeclampsia population should be considered. In the present study, among patients with preeclampsia, 22 had early-onset preeclampsia, whereas 15 had late-onset preeclampsia. Although the clinical features of early- and late-onset preeclampsia overlap, they are associated with different biochemical markers, heritability, and maternal/fetal outcomes. Future studies with larger sample sizes could allow analyses to elucidate the difference in CAVI trajectory between early- and late-onset preeclampsia. Fourth, the rate of the early-onset preeclampsia was much higher was in the present study (60%) than in general population (12%) [31]. Most of patients in our cohort were referred from primary and secondary maternity hospitals; referred patients were more likely to have early-onset and severe preeclampsia than those who were not referred to our hospital. Finally, we only used the CAVI to assess arterial stiffness. Other indices, such as the PWV or augmentation index, were not evaluated. However, the efficacy of the CAVI has been validated in numerous clinical conditions, and is correlated with other arterial stiffness markers [14, 15, 18].

## Conclusions

During 1 year of postpartum follow-up, women with preeclampsia showed a more unfavorable cardiovascular risk profile trajectory than pregnant women with normotension. However, there was no significant longitudinal difference in arterial stiffness between women with preeclampsia and normotensive pregnant women. Both groups did, however, experience a decrease in arterial stiffness, which appeared to persist after 6 months postpartum. Our findings suggest that meticulous follow-up and strict control of cardiovascular disease risk factors in women with a history of preeclampsia are advisable.

## Data Availability

Data cannot be made publicly available due to ethical restrictions set by the IRB of Seoul National University Bundang Hospital; i.e., public availability would compromise patient confidentiality and participant privacy. Please contact the corresponding author to request the minimal anonymized dataset.

## Abbreviations

AST: Aspartate transaminase
ALT: Alanine transaminase
BMI: Body mass index
BP: Blood pressure
CAVI: Cardio-ankle vascular index
HDL: High-density lipoprotein
LDL: Low-density lipoprotein
TG: Triglyceride
PWA: Pulse wave analysis
PWV: Pulse wave velocity
SNUBH: Seoul National University Bundang Hospital
IRB: Institutional review board
